# Supplementary material for: Mechanisms governing the pioneering and redistribution capabilities of the non-classical pioneer PU.1
Source: Nat Commun. 2020 Jan 21;11:402. doi: 10.1038/s41467-019-13960-2 (PMC6972792; doi:10.1038/s41467-019-13960-2)
Supplement: Supplementary file 7 — Source data [file 41467_2019_13960_MOESM7_ESM.zip › Source_Data/Figure6/Figure6B_MotifScanOutput/ETS-specific/homerResults/motif26.similar.html]

motif26

## Information for motif26

A
G
C
T
A
T
C
G
C
T
G
A
A
G
T
C
C
T
A
G
A
C
G
T
A
T
G
C
C
G
T
A
G
A
T
C
  
Reverse Opposite:  

C
T
A
G
A
C
G
T
A
T
C
G
C
G
T
A
A
G
T
C
C
T
A
G
A
G
C
T
T
A
G
C
T
C
G
A
  

|  |  |
| --- | --- |
| p-value: | 1e-38 |
| log p-value: | -8.752e+01 |
| Information Content per bp: | 1.775 |
| Number of Target Sequences with motif | 573.0 |
| Percentage of Target Sequences with motif | 7.49% |
| Number of Background Sequences with motif | 1574.8 |
| Percentage of Background Sequences with motif | 3.90% |
| Average Position of motif in Targets | 101.3 +/- 54.3bp |
| Average Position of motif in Background | 94.4 +/- 62.8bp |
| Strand Bias (log2 ratio + to - strand density) | -0.0 |
| Multiplicity (# of sites on avg that occur together) | 1.09 |
| Motif File: | file (matrix) reverse opposite |

### Similar de novo motifs found

|  |  |  |  |  |  |  |  |
| --- | --- | --- | --- | --- | --- | --- | --- |
| Rank | Match Score | Redundant Motif | P-value | log P-value | % of Targets | % of Background | Motif file |
| 1 | 0.913 | C T A G A C G T A T G C C G T A A G T C C T A G A C G T A T C G | 1e-36 | -83.449147 | 5.62% | 2.64% | motif file (matrix) |
| 2 | 0.947 | G T A C T C A G A C G T A T G C C G T A A G T C T C A G A C G T A T C G T C G A | 1e-32 | -75.240251 | 4.85% | 2.23% | motif file (matrix) |
| 3 | 0.789 | C T G A A C G T T C G A C T G A A G T C A C T G A G C T | 1e-30 | -69.153274 | 11.93% | 7.77% | motif file (matrix) |
| 4 | 0.792 | T A C G A G C T T G A C T G C A A G T C T C A G A C G T A T C G C T G A A G T C G A T C A G C T | 1e-22 | -50.755499 | 3.25% | 1.50% | motif file (matrix) |
| 5 | 0.756 | T A G C T A G C C A T G T A C G A G C T A T G C G T C A A G T C T A C G A C G T A C T G T C G A A G T C | 1e-21 | -50.539089 | 3.20% | 1.47% | motif file (matrix) |
| 6 | 0.830 | A T G C T C A G A T C G G C A T C T G A T C A G A G C T A C T G C T G A A G T C A T C G A G C T G A T C G T C A | 1e-20 | -47.265551 | 1.29% | 0.35% | motif file (matrix) |
| 7 | 0.857 | A T C G G T C A T C A G T C A G A C G T A C T G C T G A T A G C A C T G G A C T G T A C G C T A A C T G | 1e-18 | -43.528910 | 1.93% | 0.74% | motif file (matrix) |
